# Adolescence and Gender Equality in Health

**DOI:** 10.1016/j.jadohealth.2019.10.012

**Published:** 2020-01

**Authors:** Margaret E. Greene, George Patton

**Affiliations:** GreeneWorks and Promundo-US, Washington, DC; Centre for Adolescent Health, Murdoch Children's Research Institute, Parkville, Victoria, Australia; Department of Paediatrics, University of Melbourne, Royal Children's Hospital, Parkville, Victoria, Australia

Puberty brings profound shifts in identity and sense of self. The external manifestations of sexual maturity propel a young adolescent into a different set of roles and expectations. Puberty also triggers a different engagement with peers and the external world [1,2], leading to the adoption of values and aspirations a young person takes forward into adult life. Central to those values are gender norms and the sense of what it is to be a woman or man in a given culture.

Gender norms are the often unspoken rules that determine attributes and behaviors that are valued and accepted for men, women, and gender minorities [3]. They guide many life changing choices in adolescence and beyond. For girls, norms around leaving school, early marriage, and parenthood have profound effects on health and development that continue into adulthood and the next generation. In too many places, inequitable gender norms mean that a girl may not have a choice in these life altering decisions. For boys, norms likewise determine school and work decisions and how they transition into their adult sexual roles, with lasting effects on their health and well-being.

The implications of gender norms adopted in adolescence extend well beyond reproductive health and are reflected in the different health trajectories of boys and girls. For boys, injuries and injury deaths from homicide and accidents rise sharply across adolescence [3]. Although rates of adolescent self-harm and suicide attempts tend to be higher in girls, deaths from suicide are, in most places, higher among young men [4]. Substance use disorders and risks linked to alcohol, tobacco, and illicit drug use are also almost everywhere higher in young men. One consequence is that from mid-adolescence onward, boys die at higher rates than girls; in contrast, girls and women generally have higher levels of health-related disability and lower subjective well-being [5].

These sex differences in premature death, disease burden, and health risk vary across time and by place and to a large extent reflect the prevalent gender norms [3]. For girls, gender norms that emphasize girls' sexual and reproductive capacity at the expense of education, agency, and capabilities should be one target for prevention. Adolescence is a time of particular vulnerability for many mental disorders that occur much more in girls, including eating disorders, depression, and anxiety [6]. Male gender norms characterized by a need to prove themselves and dominate women and minority groups of men (e.g., those with different sexual orientations or masculine identities) similarly predict a range of health hazards related to injury, violence, and substance use in addition to violence against others including women and girls [7].

Gender norms adopted in adolescence reflect and reinforce inequitable hierarchies, whose consequences ripple forward across the life course, with health effects later in life for those young people as well as for the children of the next generation. For these reasons, adolescents should be at the forefront of research and policy action for more equitable gender norms.

In girls, research on the effects of gender norms on adolescent health has often focused on reproductive health and, in sub-Saharan Africa, vulnerability to HIV. In boys, the work has focused more on alcohol consumption and demonstrations of physical prowess through fighting and dangerous driving [8,9]. In that context, the series of papers presented in this supplement sheds new light on the determinants of gender norms in the adolescent years, their consequences for different aspects of health, and the potential for taking action to shift norms.

Meinhart et al. [10] examine the effects of peers, marital status, and educational attainment on attitudes toward tolerance of intimate partner violence in Tanzania and Nigeria. Marriage among adolescent girls and young women is associated with a greater tolerance of intimate partner violence, although they were not able to determine the direction of causality. Parents play a central role in shaping gender-related attitudes among children, illustrated in Brazil by Abdalla et al. [11], where adolescent girls were particularly susceptible to parental views: children's perception of their parents' opinion of their weight at age 11 years predicted weight control attempts at age 18 years for girls but not boys. Urbanization and Western acculturation around thinner ideal bodies have contributed to girls' greater inclination to try to lose weight. Cohen et al. [12] found that although the sons of heavier mothers in South Africa were less likely to try to lose weight, there was no association between mothers' body mass index and daughters' weight loss attempts, suggesting a generational shift among women to ideals of thinness. Chae et al. [13] explored in Zambia the role that schooling might play in changing girls' gender norms. A girl's school attendance in urban areas reinforced norms emerging from the comparatively progressive environment, whereas in rural areas, it served as a “liberalizing influence” in more conservative settings.

An article by Nagata et al. [14] illustrates the powerful effects of gender norms on body image in the U.S. A greater alignment of an individual's gender norms with the dominant views of same-sex peers was associated with greater weight loss attempts in girls and weight gain attempts in boys. The analysis by Falconi et al. [15] illustrates the malleability of gender norms and their long-term consequences by examining women's employment opportunities during World War II (WWII). Those women who took the opportunity to work intermittently during WWII and then remained in the labor force post-WWII had lower mortality across their lifetimes. Buffarini et al.'s [16] cohort study illustrates how the effects of inequitable gender norms are greatest on the most disadvantaged. Girls from low-income families fared the worst on outcomes of smoking, weight, violence, happiness, and mental health.

This supplement takes a welcome step forward in understanding the emergence and consequences of gender norms among adolescents. Yet, the articles also illustrate our limited investment in norms research. A common feature of the articles is the use of measures defined and data collected for other purposes. Many other aspects of adolescent health risks are likely to be linked to gender norms. Petroni et al. [17], for example, recently noted the links with suicide and self-harm, now, astoundingly, the major global cause of death in adolescent girls. Kapungu et al. [18] took the analysis further and made recommendations about the value of a gender lens in adolescent mental health research, prevention and service delivery, and mental health policy.

Through the inclusion of gender equity in the Sustainable Development Goals, the health needs of young women and girls have achieved greater prominence with targets around violence against women, universal access to education and sexual and reproductive health care, and equal political and economic participation [19,20]. Where such targets have been pursued, they have been accompanied by striking gains in maternal health for girls and young women, and often, the benefits extend to their children [21]. Although girls and young women have been the major focus of attention, that agenda remains far from complete. Indeed, technological change, including the rise of social media, may have introduced new risks related to the focus on physical appearance in seeking a gender ideal. For boys and young men, work on gender norms has just begun. They have much to contribute to achieving gender equality: the benefits for their own health and well-being in doing so are likely to be great.
